# A Sensor-Based and GIS-Linked Analysis of Road Characteristics Influencing Lateral Passing Distance Between Motor Vehicles and Bicycles in Austria

**DOI:** 10.3390/s26010087

**Published:** 2025-12-22

**Authors:** Tabea Fian, Georg Hauger, Aggelos Soteropoulos, Veronika Zuser, Maria Scheibmayr

**Affiliations:** 1 Research Division Transportation System Planning, Institute for Spatial Planning, Technische Universität Wien, Karlsgasse 11, A-1040 Vienna, Austria; georg.hauger@tuwien.ac.at; 2 Austrian Road Safety Board, Schleiergasse 18, A-1100 Vienna, Austriaveronika.zuser@kfv.at (V.Z.);

**Keywords:** OpenBikeSensor, lateral passing distance, cycling safety, road infrastructure, overtaking behaviour

## Abstract

**Highlights:**

**What are the main findings?**

- Using 11,399 OpenBikeSensor (OBS) overtaking records linked to Austria’s national GIS road graph (GIP), the study reveals statistically robust patterns in lateral passing distance (LPD) between motor vehicles and cyclists related to infrastructure.
- LPD is significantly higher in rural areas than in urban ones, with compliance to Austria’s 2023 legal thresholds averaging ~40% in cities (≥1.5 m) and ~19% in rural areas (≥2.0 m). Small but consistent positive correlations were found between LPD and lane width, speed limit, and functional road class.

**What are the implications of the main findings?**

- The results highlight that road geometry and network hierarchy shape overtaking safety, emphasising the need for more explicit cyclist allocation and physical separation in constrained or high-speed environments.
- The study demonstrates how sensor-based, GIS-linked nonparametric analysis can support evidence-based road design and policy evaluation regarding safe passing distances.

**Abstract:**

Lateral passing distance (LPD) when motor vehicles overtake cyclists is a key safety metric, yet infrastructure-aware evidence remains limited. This study analyses 11,399 overtaking measurements from Austria’s OpenBikeSensor (OBS) project, spatially linked to the national road graph (GIP), with urban and rural networks examined separately. LPD was treated as a continuous dependent variable, and bivariate relationships were tested using nonparametric methods: Spearman’s rho/Kendall’s tau for metric predictors (speed limit, lane width, number of lanes) and Kruskal–Wallis tests with Dunn–Holm post hoc adjustments for categorical factors (Functional Road Class, Road Configuration, Infrastructure Type). Effect sizes and confidence intervals supported substantive interpretation. LPD was higher in rural than urban contexts, with compliance to Austria’s 2023 legal thresholds averaging 40% in cities (≥1.5 m) and 19% in rural areas (≥2.0 m). Positive correlations were found between LPD and lane width, speed limit, and functional class. The findings highlight infrastructure-sensitive patterns in sensor-generated LPD and emphasise the importance of clear cyclist allocation or physical separation, especially where high speeds or spatial constraints increase close-passing risk.

## 1. Introduction

Lateral passing distance (LPD), the space left by motor vehicles when overtaking cyclists, is a key safety metric in the context of growing efforts to promote sustainable cycling mobility. Numerous international studies have investigated the influence of road design on overtaking behaviour. For instance, previous research [1,2] emphasises the role of lane width and road geometry, while dedicated cycling infrastructure has been shown to lead to greater passing distances [3,4]. Evidence from [5,6] demonstrates the risks associated with undivided roads and shared lanes, and findings from [7,8,9] confirm that lower road classes and insufficient separation correlate with higher rates of close passes. The influence of lane width has been explored in more detail in several studies. According to [10], passing distance increases by approximately 4 cm for every additional 10 cm of road width. In contrast, Ref. [11] found no significant relationship between lane width and LPD in a Swedish field study using Lidar-equipped bicycles, and [12] reported that lane width only becomes a significant factor at speed limits above 30 mph. A comprehensive risk-based analysis [13] based on more than 30,000 overtaking events in Germany emphasised that road typology, visibility, and vehicle speed interactively influence LPD. Roads with limited sight distance and high speed limits were found to result in significantly reduced passing distances, especially where cyclists lack physical separation from motor traffic. These findings highlight the importance of designing infrastructure that accounts for both spatial constraints and driver behaviour dynamics. Speed also plays an important role: [14] observed a notable increase in average passing distance on roads with higher speed limits, whereas [1] identified traffic speed as one of several significant predictors of LPD. Overtaking behaviour is also affected by factors beyond static infrastructure. Oncoming traffic, for instance, significantly reduces overtaking distance, as demonstrated in [2,5,9]. Centre line markings also matter; solid lines and rumble strips tend to constrain overtaking manoeuvres, leading to narrower gaps [10,15]. The presence of bike lanes has been widely studied with mixed results. Several works [3,4,16] report that bike lanes generally increase LPD by up to 31 cm in some contexts, whereas [17] found that overtaking distances were slightly shorter with marked lanes in Stuttgart. Similarly, Ref. [5] observed a 16 cm greater lateral distance when bike lanes were present, and [18] reported that passes below 1 m were less common on streets with bike lanes. Simulator experiments [19] showed that drivers kept 7 cm farther from the curb in the presence of a painted lane. However, no significant effect was found for bike lane presence at 30–50 mph in the UK [20], and [6] even observed reduced passing distances at 30 km/h when a bike lane was present. The type of overtaking vehicle also has a measurable impact, with SUVs tending to leave less space compared to trucks or smaller cars [8]. In addition, cyclist-related factors shape overtaking dynamics: [8] reported that cyclists wearing helmets or reflective vests were passed more closely, and that faster-moving cyclists received smaller gaps. The presence of “sharrows” (shared-lane markings) has likewise yielded mixed results, with some studies finding increased LPD due to clearer cyclist positioning [21], while others reported no effect or even reduced distances [3,22].

In the Austrian context, recent legal amendments have made LPD a central topic in traffic safety policy. In March 2023, the Austrian Straßenverkehrsordnung (StVO) [23] was revised to mandate a minimum overtaking distance of 1.5 m in urban areas and 2.0 m in rural areas when motor vehicles pass cyclists. This regulation aims to reduce the risk of collisions and improve both perceived and actual cycling safety. However, empirical data on real-world compliance and the influence of infrastructure on this behaviour remain limited. The present study addresses this research gap by linking large-scale sensor-based overtaking data with detailed GIS-derived road characteristics. Table 1 summarises the recurring determinants of LPD identified in the international literature.

## 2. Data and Methods

### 2.1. Data Sources and GIS Integration

This study is based on georeferenced measurement data from the OpenBikeSensor (OBS) project, which records overtaking manoeuvres between motor vehicles and cyclists in Austria. In recent years, community-based sensing technologies have gained relevance in cycling safety research, particularly for capturing real-world overtaking behaviour that is difficult to observe through traditional traffic monitoring methods. One such initiative is the OBS, an open-source hardware and software project developed by a distributed volunteer community to document lateral passing distances between motor vehicles and cyclists during everyday riding. The OBS consists of a bicycle-mounted unit equipped with two ultrasonic sensors and a GPS module, allowing cyclists to record overtaking events while following their normal travel routines. Before data collection, riders configure the handlebar width in the device settings, enabling the system to automatically subtract this value so that the recorded measurements reflect the true clearance between the overtaking vehicle and the outer end of the bicycle’s handlebar. During each ride, the device logs GPS tracks and all confirmed overtaking events that are explicitly confirmed by the rider via a button press on the handlebar-mounted display unit. While the full tracks are uploaded to the portal server, they remain invisible to other users unless riders explicitly adjust the visibility settings. Only the overtaking events themselves, represented as coloured points on the map, are publicly displayed. No vehicle- or person-related data about other road users are recorded or stored at any point. The system has been adopted by cycling advocacy groups in several regions, where volunteer riders contribute data to identify hazardous segments, quantify overtaking safety, and support evidence-informed infrastructure planning. Because OBS data are collected across diverse routes, bicycles, and rider profiles, the resulting datasets represent an opportunistic but wide-ranging sample of real-world interactions between cyclists and motor vehicles. In Austria, multiple OBS devices have been deployed by volunteers as part of the Radlobby Vorarlberg initiative. For this study, we used all validated overtaking records provided via the 1meter50 platform (https://www.1meter50.at, accessed on 10 December 2025), covering the period from July 2021 to October 2025.

The dataset comprises 11,399 validated overtaking events, each including high-resolution GPS coordinates and the measured LPD obtained from ultrasonic sensors mounted on bicycles. Prior to analysis, all measurements underwent systematic quality control to ensure data reliability. Records with missing spatial information or implausible values (specifically, any LPD of ≤0.30 m, which may indicate sensor misreadings or non-standard situations) were excluded. The cleaned point data were then spatially joined with the Austrian Graph Integration Platform (GIP), the national road network database, using a GIS-based nearest-neighbour approach. This spatial linkage enabled the attribution of detailed road features (e.g., speed limit, road layout, and geometric characteristics) to each individual overtaking event. To improve matching accuracy and account for spatial uncertainty in GPS measurements, all GIP line geometries were buffered by 1 m to accommodate minor GPS drift and ensure robust spatial matching. Each sensor point was then spatially joined to the nearest buffered segment. The 1 m buffer applied to the GIP road geometries does not represent the physical dimensions of a bicycle, nor does it model any bicycle envelope. Its sole purpose is to provide a spatial tolerance for correcting GPS inaccuracy and ensuring robust matching of OBS measurement points to the corresponding road segments. All lateral passing distances used in this study are derived directly from the ultrasonic sensors of the OBS device; they are not computed from, nor influenced by, the buffered geometries. This spatial operation enabled the attribution of road features (e.g., speed limits, road type, and geometry) to each overtaking measurement. To account for structural and behavioural differences, analyses were conducted separately for urban and rural road segments.

Let $P=\;\left\{p_{1},\;p_{2},\ldots ,p_{n}\right\}$ be the set of overtaking measurement points from the OBS dataset, where each $p_{i}\in R^{2}$ represents a georeferenced location of an overtaking event. Let $R=\;\left\{r_{1},\;r_{2},\ldots ,r_{m}\right\}$ be the set of line segments from the GIP road network, each represented as a polyline geometry in $R^{2}$. To account for potential GPS inaccuracy and to ensure correct matching, each road segment $r_{j}\;$ is buffered by a fixed radius ε=1 m, yielding a polygonal region:

(1) $$B_{j}=Buffer\left(r_{j},\varepsilon \right),\forall j\in \left\{1,\ldots ,m\right\}$$

**Figure 1:**
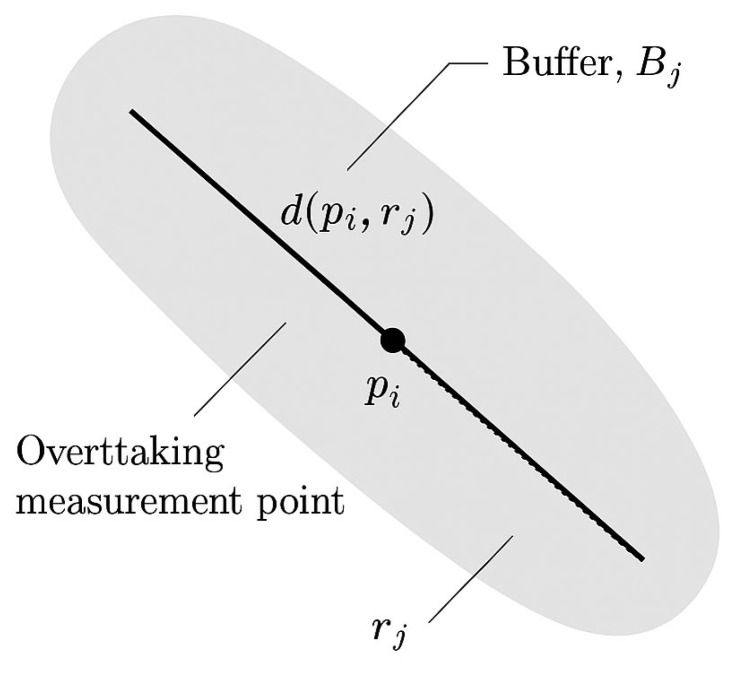
Geometric matching procedure.

We define the assignment function $f:P\to R\cup \left\{\emptyset \right\}$ such that:

(2) $$f\left(p_{i}\right)=\arg\;{min}_{r_{J}:p_{j}\in B_{j}}d\left(p_{i},r_{j}\right)$$

where $d\left(p_{i},r_{j}\right)$ is the minimum Euclidean distance from point $p_{i}$ to the geometry of $r_{j}$, and $p_{i}\in B_{j}$ ensures that only buffered segments are considered. If no $r_{j}$ satisfies $p_{i}\in B_{j}$, then $f\left(p_{i}\right)=\emptyset ,$ i.e., the point remains unassigned. This approach guarantees that only geometries within a spatial tolerance are matched and minimises attribution errors due to GPS drift or misalignment. A schematic representation of this procedure is shown in Figure 1.

### 2.2. Independent Variables

The choice of explanatory variables was constrained by the attributes available in the GIP, the national road network database. The GIP provides harmonised, nationwide information on road hierarchy, physical layout, infrastructure surface types, posted speed limits, and lane width. These variables represent the only spatially complete, consistently maintained attributes relevant to overtaking geometry across Austria. Other potentially relevant features, such as centre line markings, presence of rumble strips, or visibility constraint, are not available as structured, nationwide GIP attributes and were therefore not included in the analysis. The explanatory variables used in this study thus reflect the full set of infrastructural parameters that can be robustly linked to the sensor data on a national scale. To investigate the influence of road characteristics on LPD, two groups of independent variables were analysed:

Metric Variables: These variables describe the geometric and regulatory characteristics of the roadway at or near the point of overtaking:
  - Speed limit: Posted speed limit in kilometres per hour [km/h].
  - Lane width: Road segment width at the location of the overtaking event [m].
  - Lanes: Number of lanes in the direction of travel at the location of the event.
Categorical Variables: These variables represent structural and functional classifications of the road network, as defined by the GIP road network:
  - Functional Road Class represents the hierarchical level of the network (e.g., municipal, regional, central network), reflecting the road’s functional role within the transport system.
  - Road Configuration (derived from the GIP attribute Form of Way) describes the physical layout or division of the carriageway (whether the road is divided or undivided, includes tramway corridors, or forms shared or access spaces). This variable therefore captures the structural arrangement of the roadway.
  - Infrastructure Type (derived from the GIP attribute Basetype) indicates the specific surface or facility on which the overtaking event occurred, distinguishing, for instance, between standard roadways, multi-purpose bike lanes, dedicated bicycle lanes, sidewalks, shared pedestrian-bicycle paths, and tram tracks.

### 2.3. Statistical Methods

Before analysis, the normality of all metric variables was assessed using skewness and kurtosis statistics, as the large sample size (n = 11,399) rendered tests like Shapiro–Wilk unreliable. While the dependent variable (LPD) was approximately normally distributed, most predictor variables were not. To account for this, robust nonparametric statistical methods were applied:

- Bivariate correlation analysis: Associations between metric variables and overtaking distance were assessed using Spearman’s rho and Kendall’s Tau-b.
- Group comparison for categorical variables: The Kruskal–Wallis test was used to identify significant differences in overtaking distances between groups (e.g., different road classes). Where significant, post hoc analyses were conducted using Dunn’s test with Bonferroni and Holm adjustments.

All analyses were conducted using R. Significance levels were set at *p* < 0.05. Given the large sample size and real-world measurement conditions, a decision was made to report both effect sizes (e.g., rank-biserial correlation) and confidence intervals, to support substantive rather than solely statistical interpretation.

## 3. Results

### 3.1. Descriptive Analyses

#### 3.1.1. Speed Limit, Lane Width and Number of Lanes

Table 2 summarises the descriptive statistics for the metric variables used in the analysis, including LPD, posted speed limit, lane width, and number of lanes in direction of travel, each separately for urban and rural contexts.

**Figure 2:**
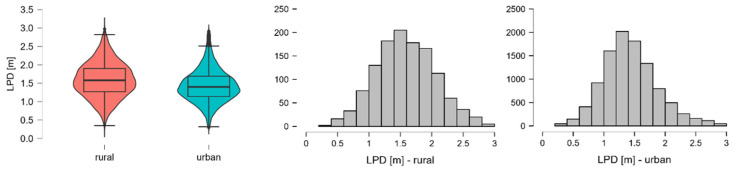
LPD across urban and rural environments, violin plots, and histograms.

The overtaking distance exhibited a higher mean in rural areas (M = 1.589 m) than in urban areas (M = 1.439 m), accompanied by a slightly greater standard deviation and interquartile range. Skewness and kurtosis values were low for both contexts (urban skewness = 0.498; rural = 0.143), indicating near-normal distribution, confirmed by acceptable levels of kurtosis (urban = 0.448; rural = −0.292). This supports the robustness of overtaking distance as a dependent variable in the subsequent statistical analysis. Figure 2 provides a visual representation of the distribution of overtaking distances across urban and rural environments, illustrating not only the central tendency and spread but also the kernel density, which confirms the slightly more symmetric distribution in rural areas.

For the explanatory variables, deviations from normality were observed. Posted speed limit showed a strong right skew in urban contexts (skewness = 0.321; kurtosis = 5.089), reflecting the dominance of low-speed zones and a few higher-speed segments. Similar skewed distributions were evident for road width (urban skewness = 1.240), which also exhibited strong kurtosis (urban = 4.437). This suggests that while most urban roads fall within a narrow width range, some exceptionally wide roads contribute to a heavy-tailed distribution. For the variable number of lanes, distributional statistics such as skewness and kurtosis are not meaningful due to its discrete and highly unbalanced nature (the majority of segments have one lane). Extremely small variance in such categorical variables can cause numerical artefacts when applying moment-based formulas, producing inflated or unstable values. Accordingly, skewness and kurtosis were not reported for this variable. The overall distributional properties of the continuous variables likewise indicated non-normality in several cases (e.g., pronounced skewness and kurtosis for lane width and speed limit in urban areas). These characteristics reinforce the decision to apply nonparametric statistical methods, as the assumption of normality is not met for multiple predictor variables.

#### 3.1.2. Compliance Modelling

To contextualise the observed LPD against the legal thresholds introduced in Austria in 2023, we applied a theoretical modelling approach based on a normal distribution. Using the empirical mean and standard deviation of the LPD data in both urban and rural environments, a normal distribution was fitted to approximate the underlying pattern of overtaking behaviour. The aim was to estimate the proportion of overtaking manoeuvres that would be expected to exceed the legal minimum passing distance purely based on this fitted distribution, assuming normally distributed variation in driver behaviour. This modelling approach enables a comparison between observed compliance rates and theoretical expectations under ideal statistical assumptions.

**Figure 3:**
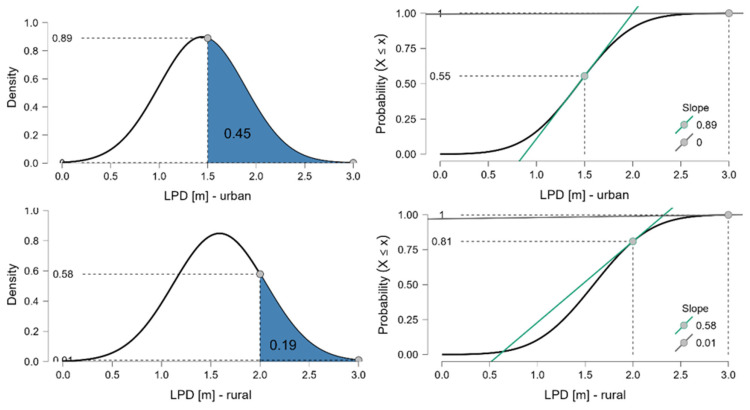
Modelled density (left) and cumulative probability (right) functions of lateral passing distance (LPD), based on fitted normal distributions using empirical means and standard deviations for urban (top row) and rural (bottom row) road segments. Vertical dashed lines indicate the legal minimum passing distance thresholds (1.5 m in urban areas, 2.0 m in rural areas). Shaded areas represent the modelled proportion of overtaking events exceeding these thresholds. Annotated values indicate cumulative probabilities at the respective thresholds. Green lines illustrate the local slope of the cumulative distribution at the threshold values. Observed compliance rates (urban: 40%; rural: 19%) are compared with modelled expectations (urban: 45%; rural: 19%).

Figure 3 presents both the density and cumulative distribution functions for urban (top row) and rural (bottom row) contexts, respectively. For urban areas, where the minimum legal passing distance is 1.5 m, the model estimates that 45% of overtaking events should exceed this threshold. However, empirical observations indicate a slightly lower rate of 40%, suggesting a shortfall in real-world compliance. In rural areas, where the minimum threshold is 2.0 m, the model predicts that 19% of events would exceed this value, which matches the observed proportion exactly. While this alignment suggests greater consistency between model and reality in rural settings, it also highlights that most overtaking manoeuvres still fall below the required threshold. These comparisons illustrate that in both settings, but especially in urban environments, a substantial share of overtaking events do not meet the legal standards. The slight mismatch in urban areas may reflect behavioural, spatial, or infrastructural barriers to compliance and should be further explored.

The normal approximation is used here for illustrative purposes only. A nonparametric ECDF approach yields nearly identical compliance estimates; the normal approximation is used only for illustration and comparability. The ECDF (Empirical Cumulative Distribution Function) represents the observed cumulative proportion of overtaking events up to any given LPD value and makes no distributional assumptions. It provides a reference distribution based on the empirical mean and standard deviation, allowing comparison between the observed compliance rates and the values expected under a symmetric distribution. This highlights that urban overtaking distances deviate slightly from the theoretical expectation, while rural compliance aligns closely with the normal approximation.

#### 3.1.3. Functional Road Class

To assess the influence of road hierarchy on LPD, we analysed the Functional Road Class separately for urban and rural road segments. Table 3 summarises key descriptive statistics for each category and area type.

**Figure 4:**
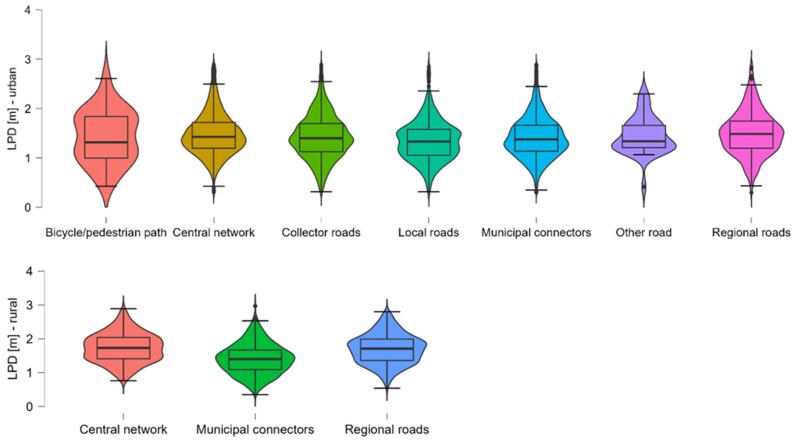
Distribution of LPD by Functional Road Class for urban and rural road segments.

Across all categories, rural segments consistently exhibit higher mean and median overtaking distances compared to their urban counterparts. This is most evident for Central network roads (rural: M = 1.734 m vs. urban: M = 1.515 m) and regional roads (rural: M = 1.700 m vs. urban: M = 1.601 m), suggesting that increased spatial availability in rural areas contributes to safer overtaking behaviour. Within urban areas, Local roads (M = 1.353 m) and Collector roads (M = 1.431 m) showed the lowest average distances, indicating a higher potential for unsafe overtaking, likely due to spatial constraints and mixed traffic conditions. In contrast, Central network and regional roads offered the greatest lateral clearance, aligning with their higher functional classification and typically wider cross-sections. In rural areas, Central network and regional roads again yielded the highest LPD values, while Municipal connectors were slightly lower (M = 1.407 m), possibly reflecting more constrained geometries or less driver awareness of passing distance regulations on lower-class roads. The variability in LPD, as indicated by standard deviation and interquartile range, is relatively stable across categories, except for the Bicycle/pedestrian path group, which shows a higher IQR (0.840). This suggests highly inconsistent overtaking behaviour in this low-sample category, warranting cautious interpretation. Several urban categories exhibit minimum passing distances below 0.5 m, which underscores persistent safety risks in dense or poorly adapted traffic environments. Figure 4 visualises the distribution of LPD across Functional Road Class for both urban and rural road segments. The violin plots summarise the density, central tendency, and variability of overtaking distances within each class. Urban categories display wider spread and higher skewness in some segments (e.g., bicycle/pedestrian paths), indicating more heterogeneous overtaking behaviour. Rural categories, in contrast, exhibit more centred and symmetrical distributions with generally higher medians, reflecting greater spatial availability.

#### 3.1.4. Road Configuration

To assess the influence of infrastructural form on LPD, the variable Road Configuration was analysed separately for urban and rural environments. Table 4 presents the descriptive statistics for each category. In urban areas, most overtaking events occurred on undivided roads (n = 7527), followed by tramway corridors and structured roadways such as divided roads (excluding motorways). In rural areas, only two categories were observed: undivided roads (n = 1099) and divided roads (n = 9). Across both settings, divided roads were associated with the highest mean LPD (urban: M = 1.677 m; rural: M = 1.709 m), while the lowest mean values were found on sidewalks (urban: M = 1.342 m) and parking areas (urban: M = 1.342 m), potentially reflecting riskier overtaking behaviour in ambiguous or shared spaces. The interquartile ranges (IQRs) suggest relatively consistent dispersion across all categories, with rural undivided roads exhibiting a slightly wider IQR (0.64 m) compared to their urban counterparts (0.53 m). These results suggest that the form of infrastructure has a meaningful effect on overtaking behaviour: more structured or dedicated road environments tend to foster greater lateral distance. However, the absence of some categories in rural areas limits broader comparison. Given the low sample size (n = 32), tramway contexts show directionally consistent but statistically unstable estimates.

**Figure 5:**
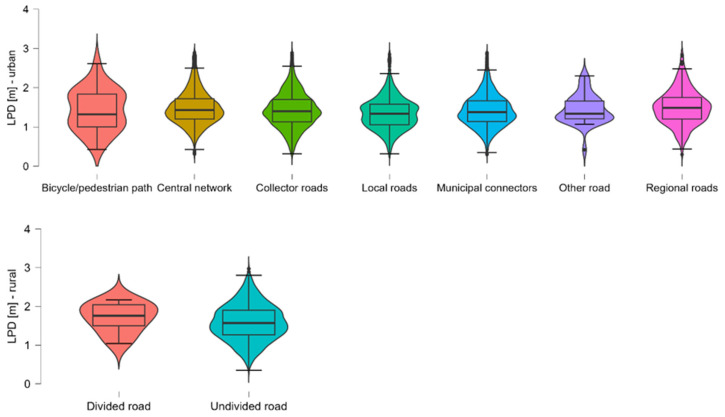
Distribution of LPD by Road Configuration for urban and rural road segments. Violin plots show the density and spread of LPD across different configuration types in both settlement contexts.

To complement the tabular overview, Figure 5 visualises the distribution of LPD across categories using violin plots for urban and rural settings.

#### 3.1.5. Infrastructure Type

To investigate the influence of different road base types on LPD, the variable Infrastructure Type was analysed separately for urban and rural areas. Table 5 summarises the descriptive statistics for each base type, focusing on the observed LPD in metres.

Urban areas: In urban contexts, most overtaking events occurred on regular roadways, with a mean LPD of 1.430 m. The lowest mean distances were observed on advisory bike lanes (1.069 m) and contraflow bike lanes (0.865 m), indicating constrained space conditions in these environments. The highest median values occurred on shared pedestrian/bike paths and sidewalks, though these categories had very low case counts (n < 25) and should be interpreted with caution. Tram track areas and standard bike lanes showed moderate mean values around 1.20–1.34 m. The variation in values, as reflected by the interquartile range and standard deviation, was also relatively narrow, except for sidewalks, where greater variability occurred.

Rural areas: In rural environments, overtaking was recorded almost exclusively on roadway segments, with a mean LPD of 1.581 m. This value closely mirrors urban roadway conditions but shows slightly higher dispersion (IQR = 0.640). No overtaking measurements were recorded on alternative base types in rural contexts. These results confirm that overtaking on regular roads dominates both spatial contexts. However, specialised cycling infrastructure (e.g., bike lanes or shared paths) tends to correlate with shorter distances in urban areas, likely due to constrained design or co-use with other traffic participants.

**Figure 6:**
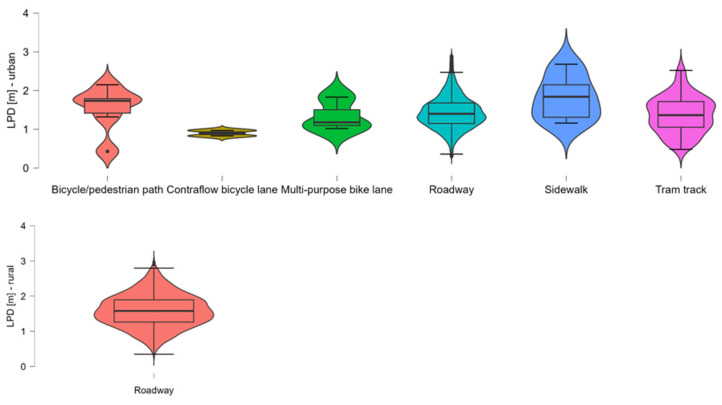
Distribution of LPD by Infrastructure Type in urban areas. Violin plots show the density and distribution of overtaking distances across infrastructure categories.

It is important to note that the imbalance in record counts across infrastructure categories reflects the underlying distribution of road types in the Austrian network. Roadway segments constitute the majority of cycling environments, whereas infrastructure forms such as contraflow lanes, multi-purpose lanes, or pedestrian–bicycle paths occur much less frequently in the network. Opportunistic sensor-based datasets naturally reproduce this structural heterogeneity rather than yielding an artificially balanced sample. The large overall sample size increases the precision of estimates for common infrastructure types; however, categories that are inherently rare in the population remain represented by small subsamples. This does not indicate sampling bias but follows directly from the low prevalence of these categories. Consequently, statistical comparisons involving rare infrastructure types should be interpreted cautiously, as their variability is more strongly influenced by limited sample size than by substantive behavioural patterns. Figure 6 visualises the distribution of LPD across urban and rural base types. Roadway segments dominate in terms of sample size but also show a moderate spread around the median (1.40 m). Notably low median LPDs are seen on contraflow bicycle lanes (0.865 m) and multi-purpose bike lanes (1.00 m), likely indicating constrained conditions. Shared spaces such as sidewalks and pedestrian/bicycle paths show greater variation, although these categories are based on smaller samples (n < 25), and results should be interpreted with caution. The consistent shape of most violins, particularly for Roadway and Tram track, suggests relatively homogeneous overtaking behaviour within these types.

In rural areas, overtaking distances are exclusively shown for the roadway base type in rural environments, reflecting the absence of dedicated cycling infrastructure in these areas. The distribution is slightly more dispersed than in urban contexts, with a wider interquartile range (IQR = 0.64 m) and higher median LPD (1.57 m), indicating greater variability and slightly safer overtaking behaviour, possibly due to increased spatial availability.

### 3.2. Bivariate Correlation Analysis

In the urban area, the correlation between the lateral overtaking distance and the posted speed limit was positive but weak (Spearman’s ρ = 0.084, *p* < 0.001), suggesting a slight tendency for overtaking distances to increase with higher maximum speed limits. A similarly weak positive correlation was observed with the total lane width (ρ = 0.062, *p* < 0.001) and the number of traffic lanes (ρ = 0.089, *p* < 0.001), indicating that wider roads and more lanes are marginally associated with greater overtaking distances. In contrast, in rural areas, the correlations were stronger. A moderate positive correlation was found between overtaking distance and speed limit (ρ = 0.217, *p* < 0.001), as well as with lane width (ρ = 0.164, *p* < 0.001). The number of lanes showed no significant correlation with overtaking distance in rural areas (ρ = –0.045, *p* = 0.136), unlike in the urban setting. In urban areas, however, the association between number of lanes and LPD is statistically significant but substantively trivial (ρ = 0.089). This effect is likely a consequence of the large sample size and the small proportion of multi-lane cross-sections in the dataset. It indicates that lane count itself contributes very little explanatory power compared to lane width or functional road class. In rural areas, where lane count varies minimally, no meaningful association was observed (ρ = –0.045, *p* = 0.136). Table 6 provides a summary of the Spearman correlation coefficients (ρ) for key infrastructural variables in relation to LPD, stratified by urban and rural contexts. The table highlights differences in the strength and significance of associations, emphasising that road width and speed limits play a more substantial role in rural areas, whereas the number of lanes is only relevant in urban settings.

### 3.3. Group Comparison for Categorical Variables

To investigate differences in Functional Road Class, Road Configuration and Infrastructure Type, separate Kruskal–Wallis tests were conducted for urban and rural environments.

#### 3.3.1. Functional Road Class

In urban areas, the test revealed a statistically significant difference in Functional Road Class ranks across 12 road types, χ^2^(11) = 69.271, *p* < 0.001. However, the effect size was small (Rank η^2^ = 0.007, 95% CI [0.005, 0.013]). Post hoc Dunn’s tests (Holm-corrected) showed that lower-function roads, such as local roads were associated with significantly lower overtaking distances compared to higher-level roads. Specifically, they differed from regional roads (*p* < 0.001, rrb = 0.207) and central network (*p* < 0.001, rrb = 0.174). Additionally, collector roads also showed significantly higher distances than local roads (*p* = 0.015, rrb = 0.105), which implies that overtaking distances tend to increase with rising network hierarchy. In rural areas, the test was also significant, χ^2^(2) = 115.901, *p* < 0.001, with a medium-to-large effect size (Rank η^2^ = 0.103, 95% CI [0.069, 0.138]). Post hoc analysis revealed that municipal connectors were associated with significantly lower overtaking distances than both regional roads (*p* < 0.001, rrb = 0.361) and central network (*p* < 0.001, rrb = 0.400). No significant differences were found between the two higher-function categories, indicating that the lowest network category in rural areas is associated with the shortest overtaking distances, whereas higher functional road class levels offer greater spatial margin for overtaking manoeuvres. The results suggest a clear relationship between road hierarchy and overtaking behaviour, with higher-function roads generally allowing larger lateral distances during overtaking of cyclists. Table 7 summarises the significant post hoc comparisons from the Kruskal–Wallis tests for Functional Road Class, highlighting only those contrasts with Holm-adjusted *p*-values below 0.05. The results emphasise that both in urban and rural settings, lower-function road categories are systematically associated with shorter LPDs. Notably, in rural areas, the observed effect sizes are considerably larger, suggesting a stronger structural influence of network hierarchy on overtaking behaviour.

#### 3.3.2. Road Configuration

In urban areas, the results revealed a statistically significant overall effect (χ^2^(7) = 37.84, *p* < 0.001, η^2^ = 0.004), although the effect size was small. Post hoc comparisons using Dunn’s test with Holm correction indicated a statistically significant difference between roads with divided roads (non-motorway) and undivided roads (*p* < 0.001), with higher overtaking distances observed for divided roads. Additionally, divided carriageways were also associated with significantly higher distances compared to parking areas (*p* = 0.013) and tram tracks (*p* = 0.047), though these differences did not remain significant after conservative correction procedures. The pairwise comparisons between all other categories showed no statistically significant differences. In rural areas, the Kruskal–Wallis test did not indicate a statistically significant difference between divided and undivided roads (χ^2^(1) = 0.99, *p* = 0.320), suggesting that the type of road separation has little to no impact on overtaking distances in non-urban contexts. Table 8 presents the results of significant post hoc comparisons for the variable Road Configuration in urban areas. Although the overall effect was statistically significant, only the contrast between divided and undivided roads remained significant after Holm correction. The findings indicate that divided roads are associated with higher LPDs. Other observed differences (e.g., versus parking areas or tram tracks) did not withstand multiple comparison adjustment and should be interpreted with caution. No significant differences were detected in rural settings.

#### 3.3.3. Infrastructure Type

To examine whether overtaking distance differed by Infrastructure Type, a Kruskal–Wallis test was conducted for urban areas. The results revealed a statistically significant difference in overtaking distances across Infrastructure Types, χ^2^(6) = 45.002, *p* < 0.001, with a small effect size (ε^2^ = 0.006). Post hoc comparisons using Dunn’s test with Holm correction showed significantly shorter overtaking distances on multi-purpose bike lanes compared to regular roadways (*p* < 0.001), shared pedestrian and cycle paths (*p* < 0.001), and sidewalks (*p* < 0.001). Additionally, contraflow cycle lanes had significantly lower overtaking distances than regular roadways (*p* = 0.052) and sidewalk (*p* = 0.028). While not formally significant after Holm correction (*p* = 0.052), the contrast suggests a substantively meaningful pattern due to the extremely low N and consistently low observed distances. There was also a significant difference between shared pedestrian and cycle paths and contraflow cycle lanes (*p* = 0.025), with contraflow lanes associated with shorter distances. Furthermore, multi-purpose bike lanes exhibited significantly lower distances than tram track (*p* = 0.006). In contrast, for rural areas, only the category standard roadway was present in the data, preventing any meaningful group comparison based on Infrastructure Type, see Table 9.

## 4. Discussion

This study examined how infrastructural and contextual factors influence LPD when motor vehicles overtake cyclists. The results confirm that road typology significantly affects overtaking behaviour. In both urban and rural contexts, infrastructure that separates cyclists from motor traffic, such as cycle lanes or wide dedicated lanes, is associated with greater overtaking distances and therefore with increased cyclist safety. Conversely, roads not originally designed for bicycle traffic, including internal access roads and undivided roads, are associated with significantly shorter overtaking distances, implying a higher risk potential. These findings are in line with previous research by [3,4,5], who highlight the protective role of dedicated infrastructure and divided carriageways. In contrast to some earlier findings, the present study identified a positive correlation between speed limit and passing distance in rural areas. This pattern suggests that higher posted speeds are typically associated with wider road geometries, which in turn allow greater lateral clearance during overtaking. The result therefore likely reflects infrastructural rather than behavioural effects, differing from studies such as [13,14], who reported reduced LPD at higher speeds under more constrained conditions. Our statistical analyses also confirm these associations more specifically: In terms of Functional Road Class, overtaking distances were significantly shorter on municipal connector roads than on regional or higher-level roads. These streets, often characterised by spatial constraints and high intersection density, appear to create conditions that foster closer overtaking. This pattern underscores the importance of context-specific infrastructure design, particularly for low-class road types and visually constrained environments, a recommendation echoed by [8,24]. For Infrastructure Type, the Kruskal–Wallis test indicated a significant association in urban areas. Post hoc comparisons showed that multi-purpose bike lanes and contraflow cycle lanes are associated with significantly reduced overtaking distances compared to standard roadways. These infrastructure types, while legally assigned to cyclists, may not provide sufficient perceptual cues to elicit cautious driver behaviour. These results align with the heterogeneous evidence in the literature, where infrastructure labelled as ‘cycling infrastructure’ does not universally lead to higher LPD, particularly when visual or physical separation is weak. Multi-purpose bike lanes, in particular, yielded the shortest overtaking distances, which is likely a result of visual ambiguity and minimal physical or symbolic separation from vehicular traffic. In contrast, shared walk- and bikeways and conventional bike lanes were associated with comparatively greater overtaking distances, supporting the hypothesis that clearly designated infrastructure increases perceived cyclist legitimacy and driver caution, even if not all differences were statistically significant after *p*-value adjustment. In rural areas, no analysis for Infrastructure Type was possible, as all observations were conducted on standard carriageways. This lack of infrastructural variation underscores the infrastructural deficit in rural cycling environments and suggests an urgent need for more diversified and context-sensitive design in rural road planning. Taken together, the findings support the broader literature emphasising the safety benefits of dedicated and context-appropriate infrastructure. However, the relatively small effect sizes observed in this study suggest that infrastructure alone may not suffice to ensure safe overtaking behaviour. Further research should examine the interplay between infrastructural cues, driver awareness, and enforcement. Moreover, awareness campaigns and regulatory adaptations may be necessary to address the gap between legal provisions and actual user behaviour.

Despite the robustness of the presented results, certain methodological and conceptual limitations should be acknowledged. First, the study is based on OBS data, which, although technically precise, depend on sensor calibration, mounting position, and GPS accuracy. These factors may introduce minor measurement variance despite the applied quality control procedures. Moreover, the data are opportunistic in nature (recorded by volunteer cyclists) thus not fully representative of all cycling contexts or rider types. Second, the study focuses exclusively on objectively measured passing distances and does not capture subjective perceptions of safety or comfort. Prior research indicates that perceived safety may diverge from physical clearance, influenced by environmental cues, noise, or driver behaviour. Integrating sensor-based measurements with on-bike perception or questionnaire data could therefore enhance the explanatory power of future studies. Third, a further methodological limitation arises from the fact that the OBS does not record motor-vehicle speeds, relative speed differences, or temporal parameters of the overtaking manoeuvre. Cyclist speed is only available at coarse temporal resolution and is not suitable for modelling dynamic interactions. OBS does not record cyclist lateral positioning before the overtaking manoeuvre; therefore, variance attributable to cyclist road placement cannot be separated from infrastructural effects. As a result, the present study cannot analyse behavioural or kinematic determinants of overtaking and focuses exclusively on infrastructural correlates of lateral passing distance. Fourth, although some variables appear to be statistically significant, the corresponding effect sizes remain mild. The large sample size increases statistical power, but the correlations themselves are small in magnitude. This indicates that infrastructure characteristics explain only a limited proportion of the variance in overtaking distance, and that overtaking behaviour is shaped by a broader set of contextual and situational factors. Finally, the analytical approach is intentionally bivariate and exploratory. Because the analytical scope is deliberately bivariate/exploratory, potential collinearity among infrastructural predictors is not modelled; however, the interpretation explicitly distinguishes infrastructural from behavioural effects. While this supports transparent interpretation, it does not control for potential interactions among variables such as speed limit, lane width, and road hierarchy. Future research could expand on this foundation using multivariate or spatially explicit models, linking technical measurement with psychological and behavioural dimensions of overtaking.

## 5. Conclusions

This study shows that overtaking safety is closely linked to road geometry and the clarity of spatial allocation. Configurations that provide clear visual or physical separation, including divided carriageways, dedicated bicycle lanes, and wider cross-sections, are consistently associated with greater lateral passing distances. In contrast, ambiguous or shared lane types, such as multi-purpose lanes and contraflow bicycle lanes, correspond to significantly shorter distances. These patterns underline the central role of cross-sectional design in shaping overtaking behaviour, particularly in constrained urban environments. These findings may inform infrastructure assessment and support evidence-based evaluations of overtaking conditions.

## Figures and Tables

**Table 1:** Key factors influencing lateral passing distance (LPD) in international research.

Influencing Factor	Mentioned in Study	Key Finding
Lane width	[1,2,10,11,12]	Wider lanes tend to increase LPD; mixed findings at low-speed limits
Number of lanes	[10,17]	More lanes can reduce LPD if they increase traffic pressure
Speed limit	[1,14]	Higher speeds may reduce LPD unless paired with sufficient road width
Cycling infrastructure	[3,4,5,6,16,17,18,19,20]	Mixed effects: most studies report increased LPD with bike lanes, but exceptions exist
Functional road class	[7,8]	Lower-class roads tend to yield shorter LPD
Road Configuration	[5,9]	Divided roads offer greater LPD than undivided ones
Infrastructure Type	[3,5]	Cycle lanes yield greater LPD than shared or pedestrian paths
Parked cars	[24]	Parked vehicles reduce available space, decreasing LPD
Urban vs. rural context	[3,8]	Rural contexts show greater variance due to diverse road typologies
Oncoming traffic	[2,9]	Oncoming traffic reduces passing distances
Centre line markings	[10,15]	Solid centre lines reduce LPD more than dashed lines
Rider appearance	[8]	Helmets and reflective vests correlate with shorter passing distances
Vehicle type	[8]	SUVs pass with less distance than trucks or passenger cars
Bicycle speed	[8]	Higher cycling speeds lead to shorter LPD
Sharrows	[1,3,22]	Mixed findings: some studies report increased LPD, others none or reduced

**Table 2:** Descriptive statistics of LPD and road characteristics (metric variables) by area type. n/a indicates that the respective statistic is not applicable, as the variable Number of lanes is discrete with only a few possible values and therefore not suitable for the calculation of skewness and kurtosis.

	LPD [m]		Speed Limit [km/h]		Lane Width [m]		Number of Lanes	
	Rural	Urban	Rural	Urban	Rural	Urban	Rural	Urban
Valid	1222	10,177	818	6686	1113	7823	1112	7536
Missing	0	0	404	3491	109	2354	110	2641
Median	1.580	1.400	80.000	50.000	6.500	7.000	1.000	1.000
Mean	1.589	1.439	77.226	47.584	6.325	7.170	1.001	1.056
Std. Deviation	0.470	0.444	12.526	9.474	1.337	2.384	0.030	0.267
IQR	0.630	0.550	10.000	5.000	1.000	2.000	0.000	0.000
Skewness	0.143	0.498	−0.214	0.321	0.353	1.240	n/a	n/a
Std. Error of Skewness	0.070	0.024	0.085	0.030	0.073	0.028	n/a	n/a
Kurtosis	−0.292	0.448	0.285	5.089	0.614	4.437	n/a	n/a
Std. Error of Kurtosis	0.140	0.049	0.171	0.060	0.147	0.055	n/a	n/a
Minimum	0.350	0.300	40.000	5.000	3.500	1.000	1.000	1.000
Maximum	2.970	2.910	100.000	100.000	13.200	22.000	2.000	4.000

**Table 3:** Descriptive statistics for LPD by Functional Road Class and area type.

Functional Road Class	Zone	N	Mean	Median	SD	IQR	Min	Max
Bicycle/pedestrian path	Urban	25	1.434	1.320	0.534	0.840	0.430	2.610
Central network	Urban	2419	1.515	1.460	0.437	0.550	0.310	2.890
Central network	Rural	275	1.734	1.730	0.429	0.630	0.760	2.890
Collector roads	Urban	1177	1.431	1.400	0.448	0.570	0.320	2.890
Local roads	Urban	542	1.353	1.335	0.410	0.520	0.320	2.850
Municipal connectors	Urban	3679	1.414	1.380	0.417	0.540	0.300	2.970
Municipal connectors	Rural	465	1.407	1.400	0.451	0.580	0.350	2.970
Other road	Urban	35	1.450	1.340	0.377	0.450	0.420	2.300
Regional roads	Urban	737	1.601	1.580	0.450	0.610	0.300	2.840
Regional roads	Rural	365	1.700	1.710	0.438	0.630	0.540	2.800

**Table 4:** LPD by Road Configuration and area type.

Road Configuration	Area	Valid N	Mean	Median	SD	IQR	Min	Max
Divided road (not motorway)	Urban	183	1.677	1.620	0.554	0.795	0.670	2.890
Divided road (not motorway)	Rural	9	1.709	1.760	0.378	0.540	1.040	2.170
Undivided road	Urban	7527	1.431	1.400	0.423	0.530	0.300	2.890
Undivided road	Rural	1099	1.582	1.570	0.468	0.640	0.350	2.970
Tramway corridor	Urban	32	1.448	1.405	0.416	0.677	0.710	2.320
Bicycle/pedestrian path	Urban	12	1.544	1.515	0.587	0.630	0.430	2.610
Sidewalk	Urban	11	1.342	1.160	0.504	0.895	0.690	1.990
Parking lot	Urban	17	1.342	1.250	0.384	0.430	0.420	2.300
Driveway to/from parking area	Urban	9	1.511	1.400	0.369	0.330	0.920	2.260
Other	Urban	13	1.585	1.600	0.260	0.530	1.240	1.980

**Table 5:** Descriptive statistics for LPD by Infrastructure Type and area type.

Infrastructure Type	Context	Valid N	Mean	Median	SD	IQR	Min	Max
Roadway	Urban	6888	1.430	1.390	0.424	0.530	0.300	2.900
Roadway	Rural	1059	1.581	1.570	0.470	0.640	0.350	2.970
Bicycle/pedestrian path	Urban	8	1.546	1.735	0.516	0.375	0.430	2.150
Sidewalk	Urban	24	1.586	1.460	0.553	0.785	0.640	2.680
Multi-purpose bike lane	Urban	35	1.069	1.000	0.425	0.465	0.490	2.550
Bicycle lane	Urban	7	1.206	1.280	0.181	0.190	0.880	1.420
Contraflow bicycle lane	Urban	4	0.865	0.855	0.081	0.085	0.780	0.970
Tram track	Urban	110	1.339	1.335	0.453	0.665	0.480	2.520

**Table 6:** Key correlations between LPD and infrastructure variables. Spearman’s ρ for urban and rural areas.

Variable	Urban Area (ρ)	*p*-Value	Rural Area (ρ)	*p*-Value
Speed limit [km/h]	0.084	<0.001	0.217	<0.001
Lane width [m]	0.062	<0.001	0.164	<0.001
Number of lanes	0.089	<0.001	−0.045	0.136

**Table 7:** Significant post hoc comparisons for Functional Road Class by area type.

Area	Comparison	Holm-Adjusted *p*	rrb
Urban	Local roads vs. regional roads	<0.001	0.207
Urban	Local roads vs. central network	<0.001	0.174
Urban	Collector roads vs. local roads	0.015	0.105
Rural	Municipal connectors vs. regional roads	<0.001	0.361
Rural	Municipal connectors vs. central network	<0.001	0.400

**Table 8:** Significant post hoc comparisons for Road Configuration (urban areas).

Comparison	Holm-Adjusted *p*	rrb
Divided road vs. undivided road	<0.001	0.246

**Table 9:** Significant post hoc comparisons for Infrastructure Type (urban areas).

Comparison	Holm-Adjusted *p*	rrb
Standard road vs. multi-purpose bike lane	<0.001	0.511
Standard road vs. contraflow bike lane	0.052	0.846

## Data Availability

The original contributions presented in this study are included in the article. Further inquiries can be directed to the corresponding author.
